# Spontaneous resolution of congenital Brown's Syndrome – a case report

**DOI:** 10.1186/1757-1626-1-7

**Published:** 2008-05-19

**Authors:** Shveta Bansal, Nishant Kumar, Ian Marsh

**Affiliations:** 1 Department of Ophthalmology, St James' University Hospital, Leeds, UK; 2 St Paul's Eye Unit, Royal Liverpool University Hospital, Liverpool, UK; 3 Department of Ophthalmology, Walton Hospital, Liverpool, UK

## Abstract

**Introduction:**

The natural history of congenital Brown Syndrome is poorly understood and documented. In our experience, few adult cases are encountered in clinical practice.

**Case Presentation:**

A case of bilateral congenital Brown syndrome is described, showing spontaneous resolution of clinical signs. Evidence of almost complete resolution over the years is also shown on serial Lees charts.

**Conclusion:**

To our knowledge, an objective record of such progressive improvement has not been previously reported. Knowledge of the natural history of Congenital Browns syndrome is useful in understanding the mechanism and also in the decision to delay surgery until full orbital growth has occurred.

## Introduction

Brown's syndrome may be congenital or acquired and is bilateral in 10% of cases.^1 ^Resolution of signs is well described in acquired, in particular inflammatory cases. The natural history of congenital Brown's syndrome however is poorly understood. Few adult cases of congenital Brown's syndrome are encountered by the clinician suggesting that partial if not complete spontaneous resolution may well occur in some individuals. Our case showed marked resolution both clinically and on Lees testing, especially in the left eye.

## Case Presentation

A five year old girl presented after her parents noticed an occasional head turn to the right. There was no significant medical, birth, developmental or family history. Visual acuities were 6/6 in the right eye and 6/9 in the left. Examination revealed no significant refractive errors and a left hypotropia measuring 6 prism dioptres (PD) for distance with the head straight. There was limited elevation of both eyes in adduction with an A pattern. Anterior segment and fundal examination was normal in both eyes.

**Figure 1:**
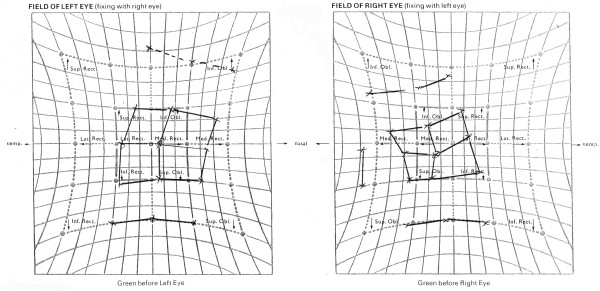
Lees Chart: Bilateral Brown's syndrome with reduced inferior oblique action and secondary overaction of the superior recti. – age 5 years.

**Figure 2:**
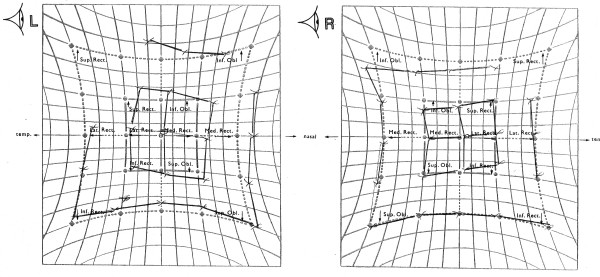
Lees Chart: Partial spontaneous resolution of bilateral Brown's syndrome – age 12 years.

The initial Lees chart showed a classical mechanical profile of a bilateral Brown syndrome (fig 1). Over the next 7 years, a spontaneous improvement in motility was noted, particularly in the right eye, both subjectively and on serial Lees charts (fig 2). At her most recent visit, the patient had a left hypotropia measuring 2 PD with no abnormal head posture. Snellens visual acuities were 6/4 in each eye and stereopsis was measured at 800 seconds of arc.

## Discussion

The limited upgaze in adduction seen in Brown's syndrome is thought to be due to an abnormality of the trochlear tendon complex [1,2]. Unlike acquired Brown's syndrome, resolution in congenital cases is poorly recorded and understood [3]. In 1962 Brown commented on the potential for spontaneous resolution [4]. Since then, improvement has even been detected with daily orthoptic exercises and unilaterally in bilateral cases [5-7]. A case of bilateral Brown's syndrome has been shown to have resolution in one eye following surgery to the fellow eye [6]. There have been reports of spontaneous resolution occurring over a period short as 7 months [5] to several years [1]. Objective evidence in the form of improved Lees Charts however has not been previously demonstrated with prior documentation of spontaneous resolution in the form of subjective observations on motility testing.

The mechanism by which resolution occurs is unclear. Reduced restriction of passage of the superior oblique tendon through the trochlear with growth of the eye has been suggested [4].

## Conclusion

The above case objectively and subjectively demonstrates spontaneous resolution in congenital Browns Syndrome. We conclude that surgical intervention in congenital cases should be delayed if possible to allow for development of the orbit as spontaneous resolution may occur. Long term observation and objective documentation of signs may aid our understanding of the natural history of Brown's syndrome.

## Competing interests

The authors declare that they have no competing interests.

## Authors' contributions

SB The lead author involved in carrying out the literature search, study design and writing the case report.

NK assisted with writing the paper, supervising and designing the study.

IM supervised the management of the case and participated in its design and approval.

All authors have been involved in approving the final manuscript.

## Consent

A copy of the written consent is available for review by the Editor-in-Chief of this journal.
